# Supplementary material for: c-Met Mediated Cytokine Network Promotes Brain Metastasis of Breast Cancer by Remodeling Neutrophil Activities
Source: Cancers (Basel). 2023 May 5;15(9):2626. doi: 10.3390/cancers15092626 (PMC10177081; doi:10.3390/cancers15092626)
Supplement: Supplementary file 1 [file cancers-15-02626-s001.zip › Supplementary Figures.pdf]

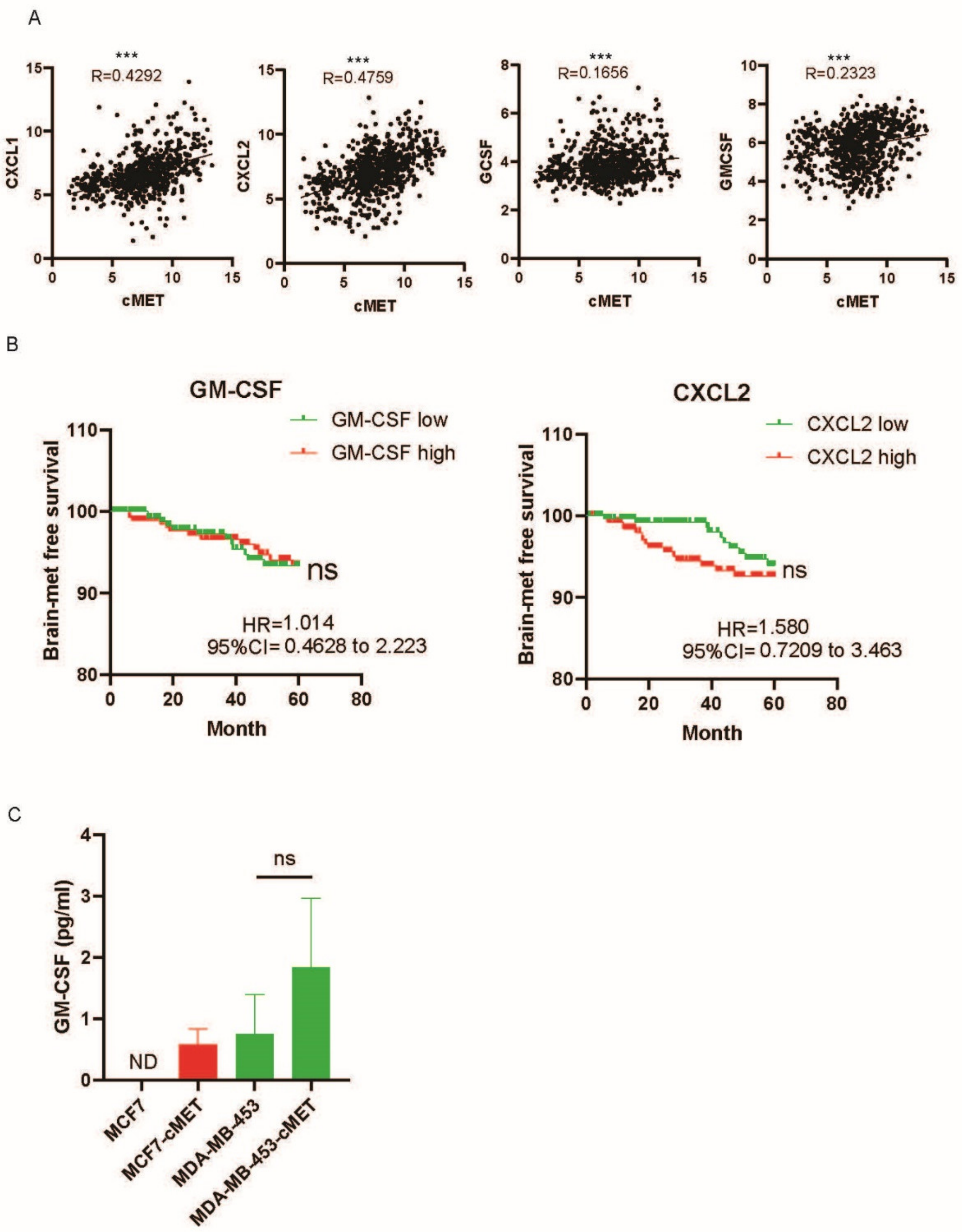

Supplementary Figure S1. c-Met pathway regulate key granulopoiesis regulators expression. **A.** Correlation between cMet and CXCL1/2 or G-CSF/GM-CSF using the combined GEO databases (GSE12276, GSE2034, GSE2603, GSE5327, and GSE14020). (Pearson correlation). **B.** Brain metastasis-free survival analyses of GM-CSF and CXCL2. **C.** ELISA of GMCSF in MCF7, MCF7-cMet, MDA-MB-453 and MDA-MB-453-cMet CM.

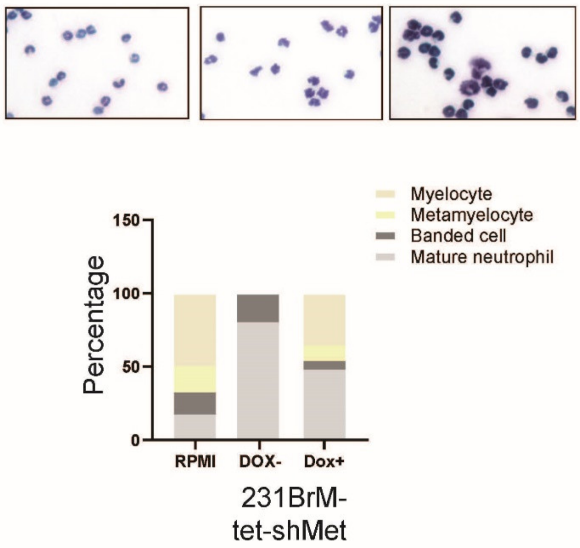

Supplementary Figure S2. c-Met pathway in cancer cells significantly promote neutrophil maturation. Upper, representative of nuclear morphology of neutrophils treated by indicated CM. Lower, quantification of percentage of different stage of neutrophil treated by indicated CM.

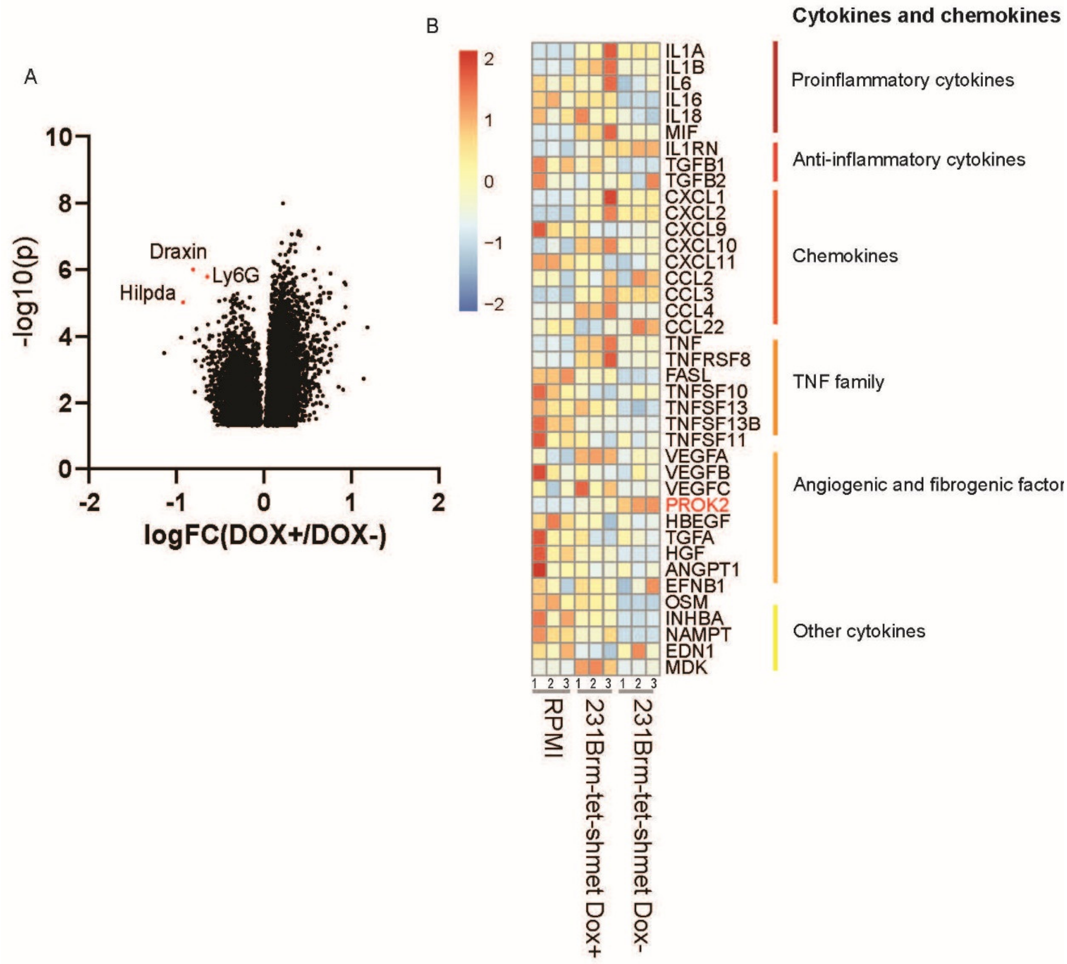

Supplementary Figure S3. c-Met pathway in cancer significantly reshape neutrophil transcriptome. A. Volcano plot of RNA sequencing of neutrophils treated by CM. Only significantly differentiated genes were show on plot. Fold change and p value between DOX+ group and DOX- group was calculated. **B.** Heatmap of key cytokines and chemokines expression from RNA sequencing after treated by indicated CM or RPMI.

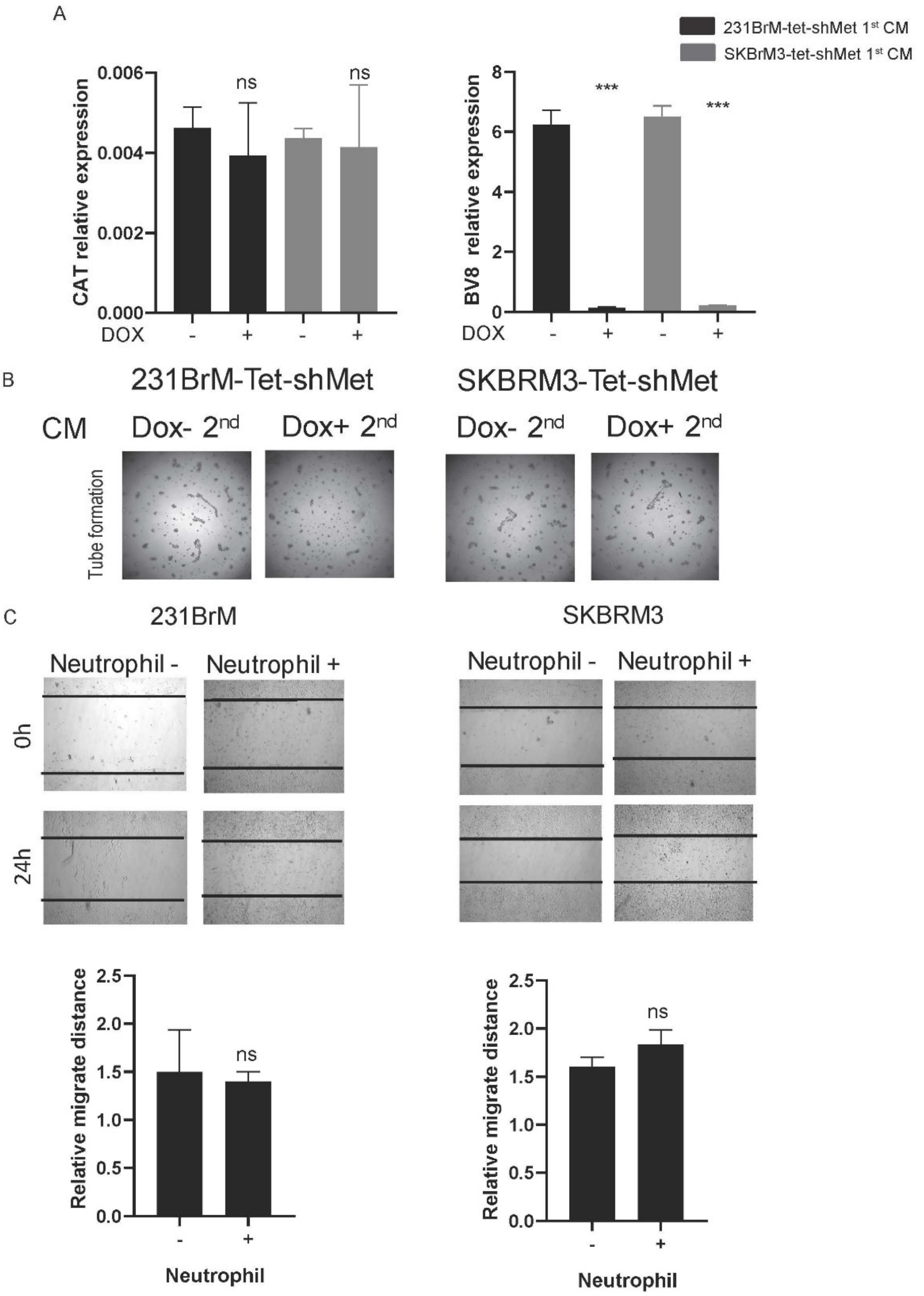

Supplementary Figure S4. N2 neutrophil does not regulate invasiveness and angiogenesis. A. Expression of CAT and BV8 was examined by qPCR. **B.** Tube formation assay of endothelial cells treated by indicated CM. **C.** Cancer cells were co-cultured with neutrophils for 48 hours before wound healing assay. Upper, representative picture of wound healing at 0 and 24 hours. Lower, quantification of migrated distance.
